# *BcGRP23*: A novel gene involved in the chlorophyll metabolic pathway that is activated by BES1 in flowering Chinese cabbage

**DOI:** 10.3389/fpls.2022.1010470

**Published:** 2022-10-19

**Authors:** Shuaiwei Zhang, Kemin Chen, Ali Anwar, Yudan Wang, Shengyi Yao, Riyuan Chen, Shiwei Song, Wei Su

**Affiliations:** ^1^ College of Horticulture, South China Agricultural University, Guangzhou, China; ^2^ Institute of Vegetables, Shandong Academy of Agricultural Sciences, Jinan, China

**Keywords:** glycine-rich protein, chlorophyll, hormone, BES1, transcriptional regulation, flowering Chinese cabbage

## Abstract

Glycine-rich proteins (GRPs) are a large family of proteins that play vital roles in cell wall remodeling, metabolism and development, and abiotic stress response. Although the functions of GRPs in cell wall remodeling have been extensively characterized, only a few studies have explored their effects on chlorophyll metabolism and hormone response. Accordingly, we aimed to determine the molecular mechanism of *BcGRP23* and its role in chlorophyll metabolism and the BRI1-EMS-SUPPRESSOR 1 (BES1) signaling pathway in flowering Chinese cabbage. The expression levels of *BcGRP23* in the leaves and stems gradually decreased with increasing growth and development of flowering Chinese cabbage, while *BcGRP23* was barely expressed after flowering. As plant growth continued, the GUS (β-glucuronidase) stain gradually became lighter in hypocotyls and was largely free of growth points. The petioles and stems of *BcGRP23*-silenced plants lost their green color, and the contents of chlorophyll a (Chl *a*) and Chl *b* were significantly reduced. Further research revealed that the expression levels of chlorophyll degradation-related genes were significantly increased in silenced plants compared with the control; however, the opposite was noted for the *BcGRP23*-overexpressing lines. The *BcGRP23* promoter sequence contains numerous hormone-responsive elements. In fact, the expression of *BcGRP23* was upregulated in flowering Chinese cabbage following treatment with the hormones indole-3-acetic acid (IAA), gibberellin (GA), 6-benzylaminopurine (6-BA), methyl jasmonate (MeJA), and brassinosteroid (BR). Treatment with BR led to the most significant upregulation. BES1, in response to BRs, directly activated the *BcGRP23* promoter. Overall, *BcGRP23* regulated the expression of chlorophyll degradation-related genes, thereby affecting the chlorophyll content. Furthermore, the expression of *BcGRP23* was significantly regulated by exogenous BR application and was directly activated by BES1. These findings preliminarily suggest the molecular mechanism and regulatory pathway of *BcGRP23* in the growth and development of flowering Chinese cabbage plants and their response to environmental stress.

## Introduction

Glycine-rich proteins (GRPs) are a class of proteins that share a high-glycine-content region and are involved in cellular stress response and signal transduction in plants (Teng et al., 2017; Czolpinska and Rurek, 2018). PtGRP1 was the first glycine-rich cell wall protein to be isolated from petunia (*Petunia hybrida*) (Condit and Meagher, 1986). More than 150 GRPs have been identified in various plant species, including maize, rice, *Arabidopsis thaliana*, and tobacco (Cornels et al., 2000; Liu et al., 2003; Zhang et al., 2014; Mangeon et al., 2016). In most plants, GRPs are classified into five groups according to the type and arrangement of their protein motifs (Mangeon et al., 2010). Members of classes I, II, and IV appear to be critically involved in the regulation of plant cells, hormone signaling, stress adaptation, and flower development (Czolpinska and Rurek, 2018). Recently, several studies have sought to determine the functions of GRPs in various plants. It was found that different environmental factors regulate the expression of GRPs, such as temperature, flooding, salinity, ultraviolet light, and hormones (Kang et al., 2013). For example, in *Arabidopsis*, the expression of *AtGRP* genes is regulated by biotic and abiotic factors (Sachetto-Martins et al., 2000). In particular, *AtGRP2* promotes seed germination and growth under salt stress in *Arabidopsis* (Fusaro et al., 2007). Both *AtGRP2* and *AtGRP7* can enhance the cold and freezing tolerance of *Arabidopsis* and rice, and GRPs that confer stress tolerance may be regulated as RNA chaperones in plants based on the efficiency of messenger RNA (mRNA) translation (Kang et al., 2013).

GRP expression is markedly tissue-specific, and its functions vary with plant growth and development (Lu et al., 2020). Analysis of 11 GRPs in different tissues and organs of *Arabidopsis* (Ma et al., 2021) revealed that *AtGRP1* is weakly expressed in all organs, while other genes had the highest expression in inflorescence cells and protoplasts. In common bean (*Phaseolus vulgaris* L.), *PvGRP1.8* was found to be mainly expressed in the xylem and cambium cells of the stem. In addition, homologous *GRP1.8* genes with the same expression pattern were found in tomato, tobacco, petunia, and soybean (Xuan et al., 2010). *OsGRP-2* is localized in the cell wall, and its promoter is specifically expressed in vascular bundles in transgenic tobacco and rice (Liu et al., 2003). Notably, some *GRP* genes are expressed in other tissues. For example, *AtGRP5* localizes to the vacuole and is preferentially expressed in developing embryonic cells (Mangeon et al., 2009; Mangeon et al., 2010). Some *GRP* genes are also specifically expressed in the roots, such as *ZmGRP3* and *ZmGRP4* in maize and *BnGRP22* in rapeseed. The expression of *BnGRP22* is induced by brassinolide at high temperatures and may enhance the stress resistance of rapeseed (Dhaubhadel and Krishna, 2008). Overall, *GRP* genes show different tissue specificities in different plants.

Photosynthesis is a fundamental element of plant growth and development. Many studies have demonstrated the crucial relationship between the photosynthetic pigment content and the growth rate of plants under abiotic stress (Xiang et al., 2016; Suhel et al., 2022). The expression of chlorophyll biosynthesis genes may alter the accumulation of chlorophyll, which causes a significant decline in plant growth and production (Xiong et al., 2018). Chlorophyll biosynthesis is catalyzed by 15 key enzymes encoded by more than 20 genes, which are highly sensitive to environmental influences (Beale, 2005). The hormone signaling pathway plays an important regulatory role in the chlorophyll biosynthesis and degradation pathways (Liu et al., 2022). Brassinosteroids (BRs) regulate plant defense systems by regulating chlorophyll content, which increases the photosynthetic capacity and antioxidant enzyme activities (Yuan et al., 2012). Exogenous application of BRs was found to enhance the expression of the chlorophyll biosynthesis genes—*RCBL*, *RCBS*, *RCA*, *SBPase*, *FBPase*, and *FBPaldolase*—under high root zone temperature in cucumber and pepper under chilly temperatures (Ding et al., 2016). Exogenous jasmonic acid (JA) induces plant leaf yellowing by regulating the expression of multiple senescence-related genes (Buchanan-Wollaston et al., 2005). Chlorophyll degradation and reduced Rubisco levels in barley (*Hordeum vulgare*) leaves led to barley leaf senescence post-treatment with exogenous JA or methyl jasmonate (MeJA) (He et al., 2002). MYC2, an essential component of the JA signaling pathway, directly interacts with ANAC019 to regulate the expression of *NYE1*/*SGR1* (STAY-GREEN protein), *NYE2*/*SGR2*, and *NYC1*, which are involved in chlorophyll degradation (Zhu et al., 2015). The ethylene signaling pathway transcription factor EIN3 promotes the degradation of chlorophyll by combining with three chlorophyll degradation genes: Non-Yellow Coloring 1 (*NYC1*), non-yellowing 1 (*NYE1*), and pheophorbide *a* oxygenase (*PAO*) (Cheng and Guan, 2014; Charoenchongsuk et al., 2015). In tomato, the expression levels of the chlorophyll degradation-related genes *PPH* (Pheophytinase) and *PAO* were upregulated after abscisic acid (ABA) treatment. Furthermore, the expression of *PAO* was negatively regulated by nordihydroguaiaretic acid (NDGA) (Mou et al., 2015). In summary, hormones are closely related to the chlorophyll biosynthesis and degradation pathways.

Although several GRPs have been identified and functionally characterized in plants, and their important role in plant development and adaptation to adversity has been revealed, relatively few studies have been conducted on the related functions of GRPs in the chlorophyll pathway in response to hormones. The flowering Chinese cabbage (*Brassica campestris* L. ssp. *chinesis* var. *utilis* Tsen et Lee) is a variant of Chinese cabbage in the *Brassica* family. It is a key vegetable crop worldwide, especially in South China, and has significant economic value (Zhang et al., 2022). In our previous study on the transcriptome of propiconazole treatment, *BcGRP23* was significantly downregulated under exogenous substance stress in the flowering Chinese cabbage (**Supplementary Figure S6**). *GRP23* was hypothesized to be involved in the regulation of the hormone-induced chlorophyll pathway and to play an unknown biological function in the growth regulation and abiotic stress response of flowering Chinese cabbage. Accordingly, *BcGRP23* was isolated from flowering Chinese cabbage plants to characterize its function and explore the molecular mechanisms whereby it regulates the growth of flowering Chinese cabbage. The findings of this study will lay the foundation for an in-depth understanding of the molecular mechanisms affecting plant growth and GRP responses to the environment.

## Materials and methods

### Plant materials and growth conditions

The flowering Chinese cabbage cultivar ‘Youlv 501’ was obtained from the Guangzhou Academy of Agricultural Science and cultivated in a greenhouse at the College of Horticulture, South China Agricultural University. Wild-type (WT) and *BcGRP23* transgenic plants were grown in the temperature range 23°C–25°C, with 16-h light (100 µmol m^−2^ s^−1^) and 8-h dark. Knockdown lines generated by transient virus-induced gene silencing (VIGS) were cultured at 22°C/20°C (day/night). The other environmental conditions were the same as those listed above. Different tissue parts of normal growing flowering Chinese cabbage seedlings were sampled at different growth stages (cotyledon, three-leaf, six-leaf, initial flowering, and full-bloom stages) for quantitative real-time PCR (qRT-PCR). *A. thaliana* Columbia-0 (Col-0) was used for the genetic transformation of *BcGRP23*. Transgenic lines and WT *Arabidopsis* plants were maintained normally in a growth chamber (23°C–25°C, with a light cycle of 16-h light and 8-h dark).

### Cloning and bioinformatics analysis of *BcGRP23*

Total RNA was extracted from the leaves and stems of flowering Chinese cabbage using the Eastep^®^ Super Total RNA Extraction Kit (Promega, Shanghai, China) according to the manufacturer’s instructions. First-strand complementary DNA (cDNA) was synthesized and subjected to reverse transcription using the PrimeScript II 1st Strand cDNA Synthesis Kit (Takara, Dalian, China). The coding sequences (CDS) of *BcGRP23* isolated from flowering Chinese cabbage were cloned using gene-homologous primers with reference to the *Brassica rapa* genome (**Supplementary Table S1**). The nucleic acid and promoter sequences of Chinese cabbage *BrGRP23* (LOC103853913) were obtained from the National Center for Biotechnology Information (NCBI) (http://www.ncbi.nlm.nih.gov). Amino acid (aa) prediction of *BcGRP23* was performed using EditSeq software (DNAStar Inc., Madison, WI, USA). Multiple sequence alignment was performed using MEGA7.0, and a phylogenetic tree was constructed using MEGA7.0 and the neighbor-joining algorithm with 1,000 bootstrap replicates (Kumar et al., 2016). Promoter *cis*-acting regulatory element searches were conducted on promoters using the PlantCARE software (http://bioinformatics.psb.ugent.be/webtools/plantcare/html/).

### Subcellular localization

The full-length *BcGRP23* CDS without a stop codon was cloned into the pBI121-GFP vector and fused with the gene sequence of the green fluorescent protein (GFP). The primers used for cloning are listed in **Supplementary Table S2**. Onion epidermal cells were first infiltrated with the *Agrobacterium tumefaciens* strain GV3101 and transformed with the corresponding constructs. The GFP signal was captured using a fluorescence microscope (Axio Imager D2, Zeiss, Oberkochen, Germany). All assays were repeated three times.

### Quantitative real-time polymerase chain reaction

Total RNA was isolated from the leaves and petioles of WT and *BcGRP23* transgenic plants. Thereafter, qRT-PCR was performed using a SYBR Premix EX Taq kit (Takara, Dalian, China) on a LightCycler 480 Real-Time PCR instrument (Roche Diagnostics, Rotkreutz, Switzerland). Three biological and three technical replicates were used for each sample. The glyceraldehyde-3-phosphate dehydrogenase (*GAPDH*) gene (XM_033273046.1) was used as the internal control. The primers used for qRT-PCR are listed in **Supplementary Tables S2, S3**.

### Virus-induced gene silencing assay

The *BcGRP23* CDS was cloned into the pTRV2 plasmid to generate the silencing vector, pTRV2:*BcGRP23*. The specific experimental procedure is described elsewhere (Chen et al., 2011). The pTRV2:*BcGRP23* and pTVR1 vectors were co-infiltrated into the cotyledons of young flowering Chinese cabbage seedlings. Two weeks after infection, the second true leaf was collected from each plant, and RNA was extracted for qRT-PCR to determine the efficiency of gene silencing. Plants co-infiltrated with pTRV2 and pTRV1 were used as negative controls (Wang et al., 2022). The primers used for the VIGS assay are listed in **Supplementary Table S2**.

### Agrobacterium-mediated transformation in flowering Chinese cabbage

Transgenic flowering Chinese cabbage plants overexpressing *BcGRP23* were obtained using the PBI121-BcGRP23-GFP vector. Cotyledon transformation of the ‘Youlv 501’ cultivar was performed using our laboratory method (Wang et al., 2022). Positive transgenic plants were identified *via* qRT-PCR of *BcGRP23* in the leaves and petioles.

### *Arabidopsis thaliana* transformation

The *BcGRP23* promoter was cloned into the pCAMBIA1391-GUS vector to obtain the pCAMBIA1391-*proBcGRP23*-GUS recombinant plasmid, which was introduced into the *A. tumefaciens* strain GV3101. The primers used for cloning are listed in **Supplementary Table S2**. *Arabidopsis* Col-0 transgenic plants expressing pCAMBIA1391-*proBcGRP23*-GUS were generated using the floral dip method. Positive plants were screened on Murashige–Skoog (MS) medium containing 25 mg/L hygromycin and were identified using PCR.

### Histochemical GUS staining analysis

T2 generation transgenic *Arabidopsis* plant tissues from different parts of the plant at different growth stages were selected, placed in an appropriate amount of β-glucuronidase (GUS) staining solution at 37°C for 12 h, and then decolorized with 50%, 70%, and 95% ethanol in a gradient manner until complete. The samples were stored in 70% ethanol for examination of their expression characteristics.

### Exogenous hormone treatments

Seedlings of transgenic *Arabidopsis* lines in the cotyledon stage were treated with 50 μmol L^−1^ ABA, indole-3-acetic acid (IAA), gibberellin (GA), 6-benzylaminopurine (6-BA), salicylate (SA), MeJA, and BR and then immersed in water as a control. The concentration of the hormone treatments was the optimal concentration determined during the pre-experiment. After 3 h of treatment, histochemical GUS analysis was performed using the same method described above. Three-week-old seedling leaves of flowering Chinese cabbage (WT) were sprayed with 100 μmol L^−1^ ABA, IAA, GA, 6-BA, SA, MeJA, or BR (treated with water as a control). After 6 h, the samples were collected and the transcript levels of *BcGRP23* determined using qRT-PCR.

### Yeast one-hybrid assays

Yeast one-hybrid (Y1H) assays were conducted using the Matchmaker One-Hybrid System (Clontech, Palo Alto, CA, USA) (Wu et al., 2022). The 572-bp region surrounding the *BcGRP23* promoter sequence was synthesized with the *Knp*I and *Sal*I flanking restriction sites and then cloned into a *Kpn*I/*Sal*I-digested pAbAI bait vector. For expression in yeast, the cDNA of BcBES1 was cloned into the pGADT7 prey vector. *Saccharomyces* Y1H Gold (Clontech, Palo Alto, CA, USA) was used as the host. We first digested the pAbAI-*proGRP23* vector using *Bst*BI), and then integrated it into Y1H Gold. The bait strain (Y1H-pAbAI-*proGRP23*) was screened for self-activated on synthetically defined (SD)/−Ura with aureobasidin A (AbA*). Transformation was confirmed *via* growth on SD/−Leu medium (Clontech, Palo Alto, CA, USA) containing AbA (Yeasen, Shanghai, China). The primers used are listed in **Supplementary Table S2**.

### Dual-luciferase reporter assays using *N. benthamiana* leaves

For analyses of the transcriptional activity of BcBES1 to *BcGRP23* in *Nicotiana benthamiana* leaves, the CDS of BcBES1 was amplified by PCR and inserted into the pGreenII 62-SK vector to generate the effector. To generate the reporter constructs, the promoter fragments of *BcGRP23* were inserted into the pGreenII 0800 vector. The empty pGreenII 62-SK vector was used as a control. The constructs were then introduced into tobacco plants *via Agrobacterium*-mediated transformation. Firefly luciferase (LUC) and *Renilla* (REN) activities were measured using a dual-luciferase reporter assay kit (Promega, Madison, WI, USA). The relative ratio of LUC to REN was calculated to represent the expression of the reporter genes. The experiment was performed three times, with each sample also tested three times. The primers used for these vectors are listed in **Supplementary Table S2**.

## Results

### Sequence analysis and subcellular localization of *BcGRP23* in flowering Chinese cabbage

**Figure 1:**
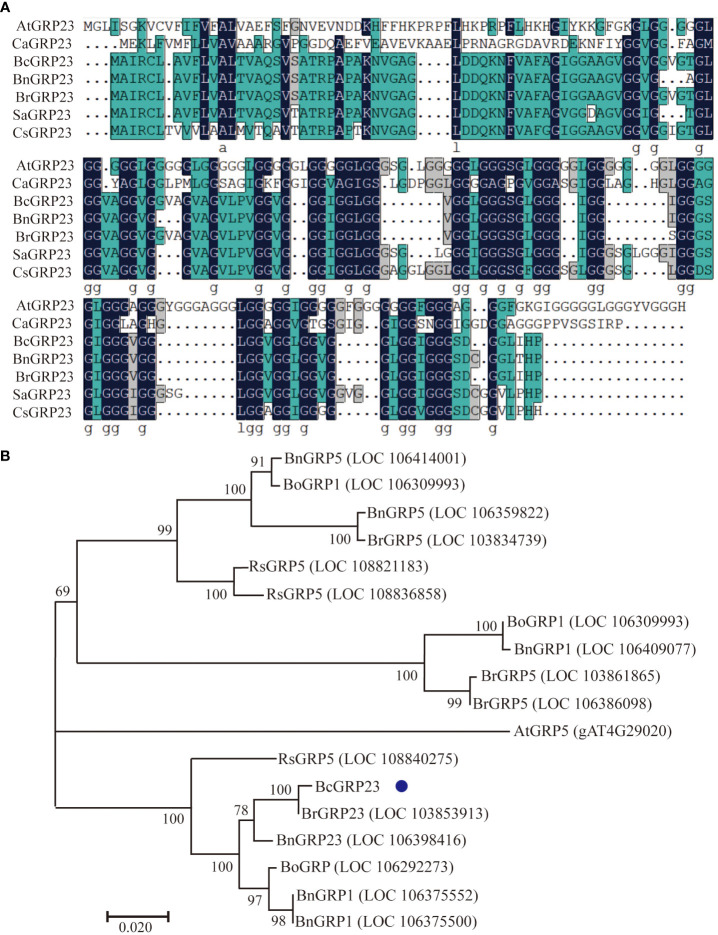
Protein sequence alignments of *BcGRP23* in flowering Chinese cabbage and other species. **(A)** Multiple sequence alignment performed using MEGA7 software. Amino acids with *blue background* are highly conserved. The sequences were markedly similar between *Brassica rapa*, *Cucumis sativus*, *Brassica napa*, *Sinapis alba*, *Camelina sativa*, and *Arabidopsis thaliana*. **(B)** Phylogenetic analysis of the glycine-rich protein (GRP) sequences of different plant species performed using the neighbor joining method in MEGA7, with 1,000 bootstrap replicates. *Bo*, *B. oleracea*; *Bn*, *B. napus*; *Bc*, *B. campestris*; *Rs*, *Raphanus sativus*.

Specific primers were designed for the *BcGRP23* gene (CDS) based on the sequence of the *BrGRP23* gene from Chinese cabbage. The *BcGRP23* gene was then isolated from flowering Chinese cabbage and confirmed using PCR. Based on the sequencing results, a *BcGRP23* open reading frame (ORF) with 444 bp was obtained (**Supplementary Figure S1**). The ORF of *BcGRP23* was identified to encode 147 amino acids (**Supplementary Figure S1A**). Subsequently, the aa sequence alignment of GRP23 homologous proteins from *Arabidopsis*, Chinese cabbage, and flowering Chinese cabbage was performed using MEGA7 software (**Figure 1A**). The results showed that the homology of the *BcGRP23* and *BrGRP23* (LOC103853913) aa sequences was 99.32%, and both contained multiple GGX or GGGX tandem repeats. In contrast, the homology between *BcGRP23* and *AtGRP23* (AT2G32690) aa sequences was only 29.85%, with *AtGRP23* containing multiple tandem repeats of GGGX and GGGGGX.

**Figure 2:**
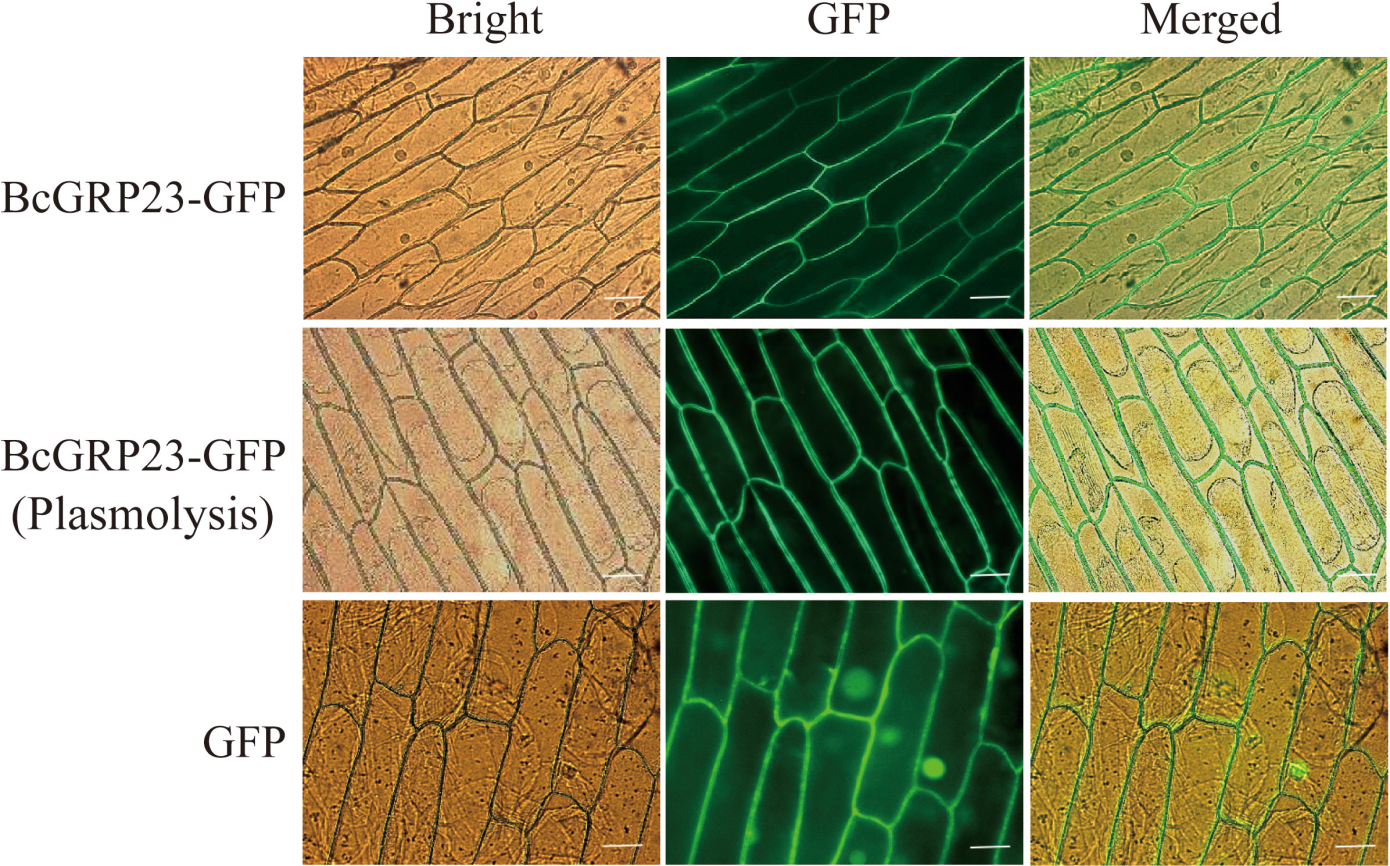
Subcellular localization of *BcGRP23*. The vectors 35S::GFP (pBI121-GFP, positive control) and 35S:BcGRP23-GFP (pBI121-BcGRP23-GFP) were injected into onion epidermal cells and visualized with a fluorescence microscope after 24 h of bombardment. *Bar*, 25 μm.

To explore the evolutionary relationships among the *BcGRP* genes, a maximum likelihood (ML) phylogenetic tree was constructed according to the *GRP* protein sequences from different species (**Figure 1B**). The evolutionary relationship analysis indicated that *BcGRP23* is closely related to GRP23 in other cruciferous species (*B. rapa* and *Brassica napus*). In fact, *BcGRP23* showed 99.95% homology with *BrGRP23* and 94.59% homology with *BnGRP23*, whereas *BcGRP23* showed 92.57% homology with *RsGRP5*. *BcGRP23* had a homology of 90.13% and 84.75% with BnGRP1 and BnGRP5, respectively (**Figures 1A, B**). When the pBI121-BcGRP23-GFP plasmid was transformed into the onion epidermis, an obvious GFP signal was observed in the cell wall of the plasmolysis onion epidermis, but not on the cell membrane (**Figure 2**). This result indicates that *BcGRP23* was localized in the cell wall.

### Expression analysis of *BcGRP23* in different tissues at various developmental periods

**Figure 3:**
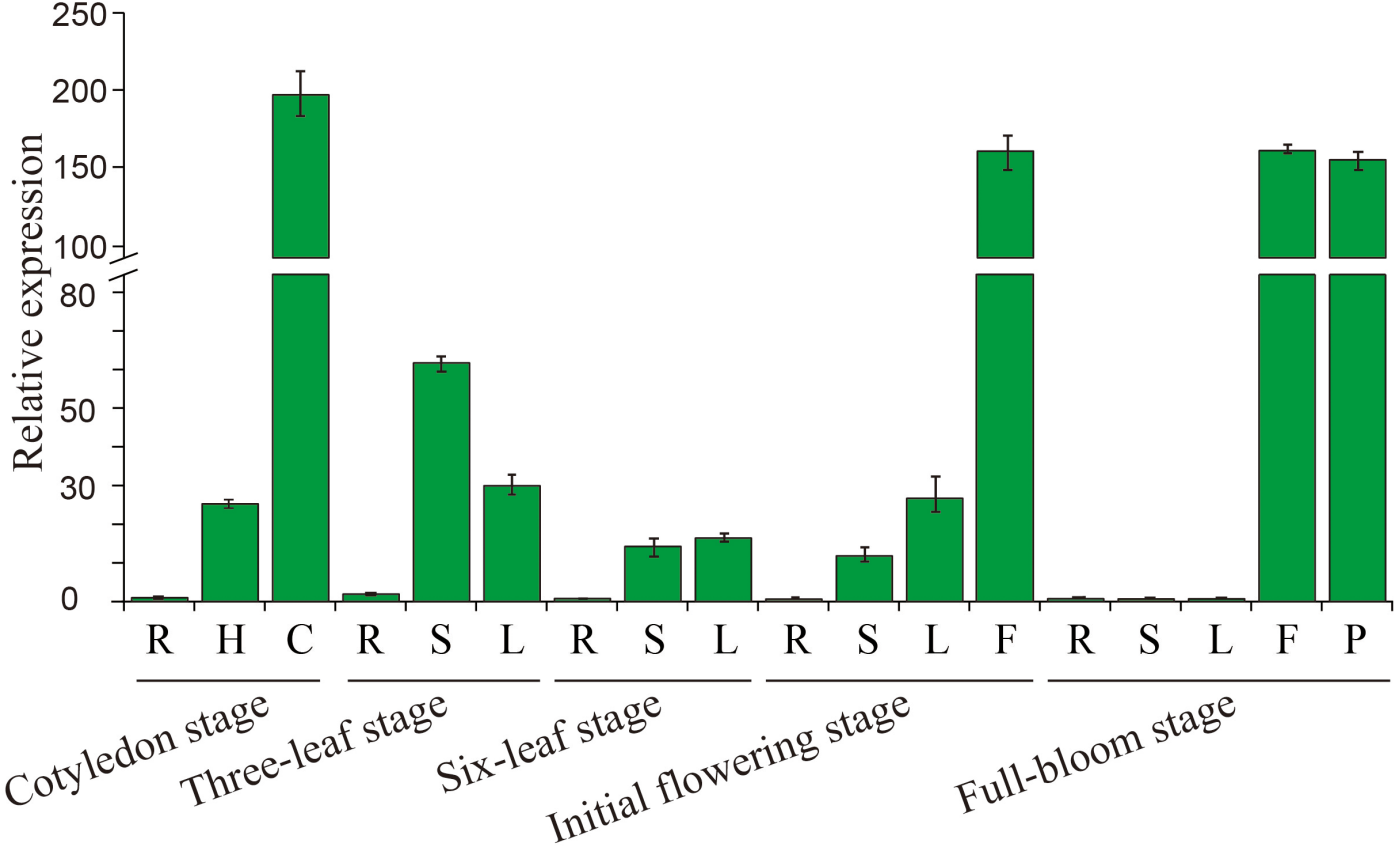
Expression analysis of *BcGRP23* in different tissues at various developmental stages. *R*, root; *H*, hypocotyl; *C*, cotyledon; *S*, stem; *L*, leaf; *F*, flower; *P*, pod. *Bar on top of the columns* represents the standard deviation.

To assess the tissue-specific expression of *BcGRP23*, qRT-PCR was performed (**Figure 3**). Noticeable differences were identified when the transcript levels of *BcGRP23* in different organs of flowering Chinese cabbage were assayed. The expression level of *BcGRP23* in the roots was low during the entire growth period; however, in the cotyledon stage, flowers in the initial flowering stage and the flowers and seeds in the full-bloom stage were the highest, with values 197-, 159.4-, 159.7-, and 153.6-fold higher than those of the roots in the cotyledon stage, respectively. When the stems at different stages were analyzed, the highest expression of *BcGRP23* was found at the three-leaf stage, with a value 2.4-fold higher than that at the cotyledon stage; this level was significantly higher than that in the six-leaf stage and the initial flowering stems. Notably, almost no expression was observed in flowering stems. The expression level of *BcGRP23* in leaves was the highest in the cotyledon stage, with a value 6.3-fold higher than that of the leaves in the three-leaf stage. No significant difference in the expression of *BcGRP23* was found in the three-leaf stage, six-leaf stage, and the initial flowering stage. Furthermore, *BcGRP23* was not expressed in the flowering stage.

### *BcGRP23* silencing accelerates chlorophyll degradation in flowering Chinese cabbage

**Figure 4:**
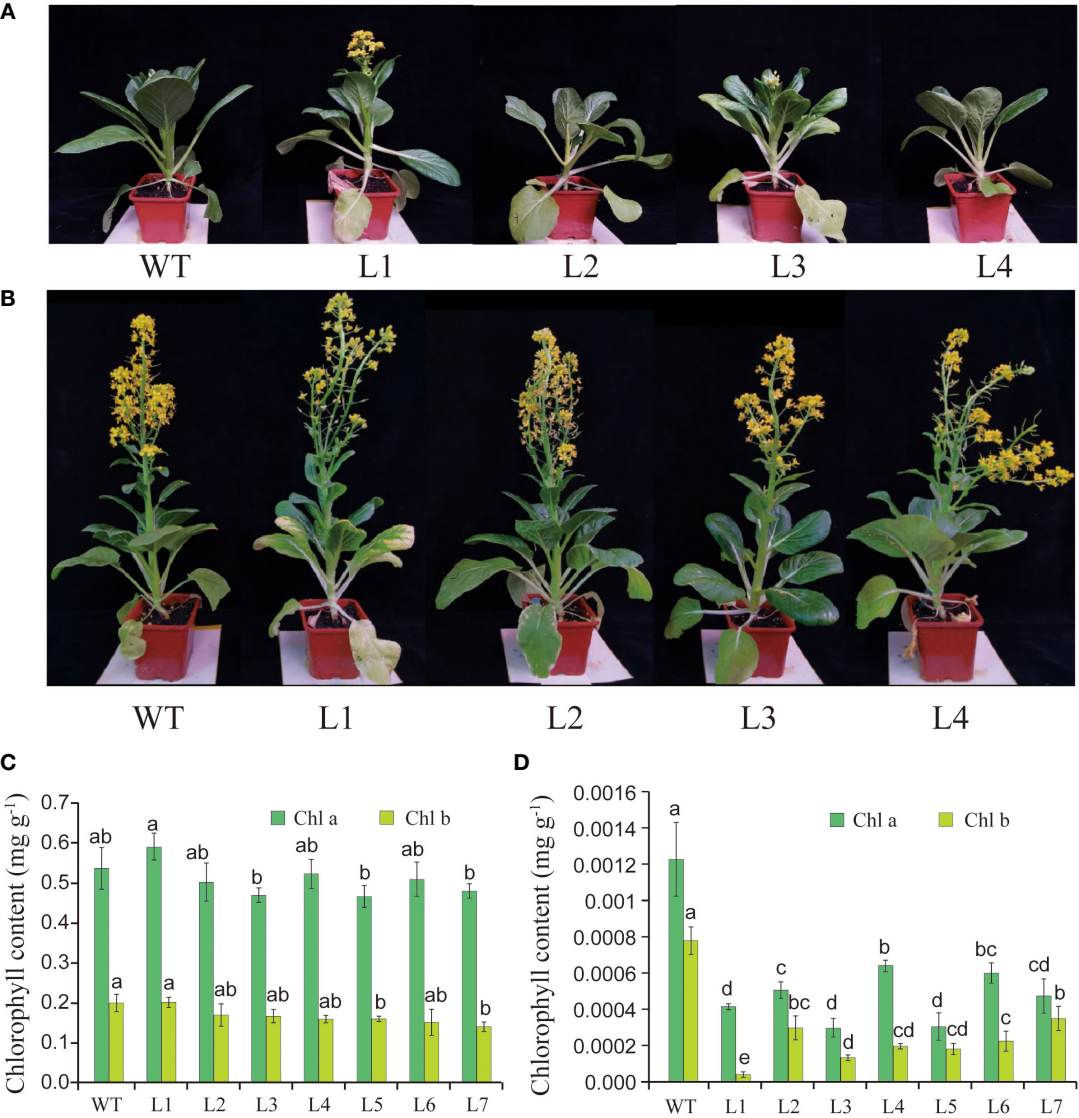
**(A, B)** Effect of *BcGRP23* knockdown on the phenotypes of flowering Chinese cabbage at the initial flowering stage **(A)** and the full-bloom stage **(B)**. Transgenic lines carrying the empty pTRV2 vector (EV) were used as wild type (WT). *L1*–*L4* represent pTRV2:*BcGRP23*. **(C, D)** Chlorophyll contents of the leaves **(C)** and petioles **(D)** in the *BcGRP23* knockdown lines. *L1*–*L7* represent different *BcGRP23* knockdown lines. *Error bars* represent standard errors. *Different lowercase letters* indicate significant differences at the *p* < 0.05 level.

To explore the biological function of *BcGRP23*, the phenotypes of the pTRV2:*BcGRP23* knockdown lines were analyzed. Most interestingly, the pTRV2:*BcGRP23* knockdown lines showed whitening of the stems and petioles compared with the WT (pTRV2 empty vector) in the initial flowering stage (**Figure 4A**). In fact, the stems and petioles of the pTRV2:*BcGRP23* knockdown lines lost their green color. However, the stems, petioles, and flowers of the upper half of the knockdown lines were not significantly different from those of the WT after entering the flowering and fruiting stages (**Figure 4B**). Overall, the silencing of *BcGRP23* could lead to white stems and petioles; however, only the stems and petioles of the lower half turned white during the flowering and fruiting stages. This result may be due to the timeliness of the VIGS knockdown lines. The whitening phenomenon may be related to the decrease in chlorophyll content. Therefore, the contents of chlorophyll a (Chl *a*) and Chl *b* in the leaves and petioles were determined in the pTRV2:*BcGRP23* knockdown lines and in WT. Chl *a* and Chl *b* in the leaves of the pTRV2:*BcGRP23* knockdown lines were not significantly different from those of the WT (**Figure 4C**). However, the contents of Chl *a* and Chl *b* in the petioles of the pTRV2:*BcGRP23* knockdown lines were significantly lower than those in WT plants (**Figure 4D**). Thus, the silencing of *BcGRP23* could reduce the contents of photosynthetic pigments such as Chl *a* and Chl *b*, which may be the reason for the whitening of the petioles and stems.

### Silencing of *BcGRP23* significantly induces the expression of chlorophyll degradation-related genes in flowering Chinese cabbage

**Figure 5:**
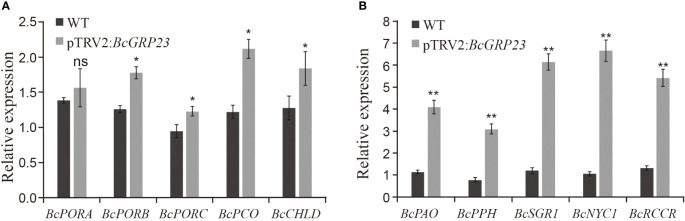
Expression of the genes related to chlorophyll biosynthesis **(A)** and degradation **(B)** in the petioles of the pTRV2:*BcGRP23* knockdown lines. *Significant difference at the *p* < 0.05 level; **significant difference at the *p* < 0.01 level; *ns*, no significant difference (Student’s *t*-test).

To further explore the role of *BcGRP23* in chlorophyll biosynthesis and degradation, the transcript levels of five chlorophyll biosynthesis-related genes (*BcPORA*, *BcPORB*, *BcPORC*, *BcCAO*, and *BcCHLD*) and four chlorophyll degradation genes (*BcPAO*, *BcPPH*, *BcNYC1*, *BcRCCR*, and *BcSGR1*) were measured in pTRV2:*BcGRP23* and WT lines. Compared to the WT, the expression levels of the chlorophyll biosynthesis-related genes—*BcCAO*, *BcPORC*, *BcCHLD*, and *BcPORB*—increased by 1.73-, 1.29-, 1.44-, and 1.41-fold, respectively, in the petioles of the knockdown lines (**Figure 5A**). However, the expression level of *BcPORA* did not differ significantly.

The expression levels of the chlorophyll degradation genes were significantly increased in the pTRV2:*BcGRP23* knockdown line compared with the WT. Furthermore, the magnitude of the upregulation of these genes was greater than that of the chlorophyll biosynthesis-related genes. Among them, the expression levels of *BcNYC1*, *BcPAO*, *BcPPH*, *BcRCCR*, and *BcSGR1* were upregulated by 6.26-, 3.59-, 4-, 4-, and 5.13-fold, respectively, in the petioles (**Figure 5B**). Therefore, the silencing of *BcGRP23* significantly upregulated the expression of the chlorophyll degradation-related genes in the petioles, accelerated chlorophyll degradation, and whitened the petioles of flowering Chinese cabbage.

### *BcGRP23* overexpression might induce an increase in chlorophyll accumulation by downregulating chlorophyll degradation-related genes

**Figure 6:**
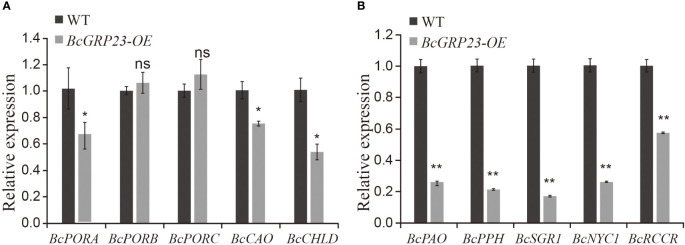
Expression of the chlorophyll biosynthesis-related **(A)** and degradation-related **(B)** genes in the petioles of *BcGRP23*-overexpressing plants. *Significant difference at the *p* < 0.05 level; **significant difference at the *p* < 0.01 level; *ns* indicates that the difference between the two lines is not statistically significant (Student’s *t*-test).

To explore the role of *BcGRP23* overexpression in flowering Chinese cabbage, the cotyledons of flowering Chinese cabbage plants were infected *via Agrobacterium*-mediated transformation. Four positive transgenic plants were obtained by screening seeds of the T0 generation. We determined the expression of *BcGRP23* in the transgenic lines and found that the expression in L1 was significantly higher (i.e., 14.7-fold higher) than that in WT plants. The expression levels of *BcPORB* and *BcPORC* in the petioles were not significantly different between the overexpressing and WT plants. However, the levels of *BcPORA*, *BcCAO*, *BcCHLD*, *BcPAO*, *BcPPH*, *BcNYC1*, and *BcRCCR* showed a significant downward trend. Among them, the expression levels of *BcPORA*, *BcCAO*, and *BcCHLD* were found to decrease by 35%, 25%, and 46%, respectively, in the overexpression line compared with the WT (**Figure 6A**). In addition, the expression levels of the chlorophyll degradation genes (*BcPAO*, *BcPPH*, *BcNYC1*, *BcRCCR*, and *BcSGR1*) decreased by 73%, 78%, 82%, 74%, and 42%, respectively, in the overexpression line compared to the WT (**Figure 6B**). Altogether, these results suggest that *BcGRP23* could inhibit the expression of the chlorophyll biosynthesis and degradation-related genes, with a more significant inhibitory effect on the degradation-related genes than the biosynthesis-related genes.

### Cloning and functional analysis of the *BcGRP23* promoter

Analysis of the *cis*-acting elements contained in the promoter region of *BcGRP23* revealed that the *BcGRP23* promoter contains different hormone-responsive elements (ethylene-, ABA-, and brassinolide-responsive elements, GA-related elements, MeJA-responsive elements, cytokinin response elements, and salicylic acid-inducible response elements) and some adversity stress response elements (**Supplementary Table S1**). Such results suggest that *BcGRP23* may interact with these hormone signaling pathways when exposed to various hormones and abiotic stresses. The *BcGRP23* promoter sequence also had a large number of light regulatory elements, some chlorophyll-related regulatory elements (CPBCSPOR and GATABOX), and different tissue-specific expression regulatory elements [guard cell-specific gene expression elements, mesophyll-specific regulatory elements, one each of multifunctional transcription factors related to flowering gene transcription, pollen late gene promoter, flower organ-specific element, DOF (DNA binding with One Finger) gene binding site, tissue-specific expression-related *cis*-elements, seed-specific expression elements, and other organ-specific regulatory elements] (**Supplementary Table S1**). Therefore, the expression of *BcGRP23* may be tissue-specific and related to the chlorophyll pathway in flowering Chinese cabbage plants.

**Figure 7:**
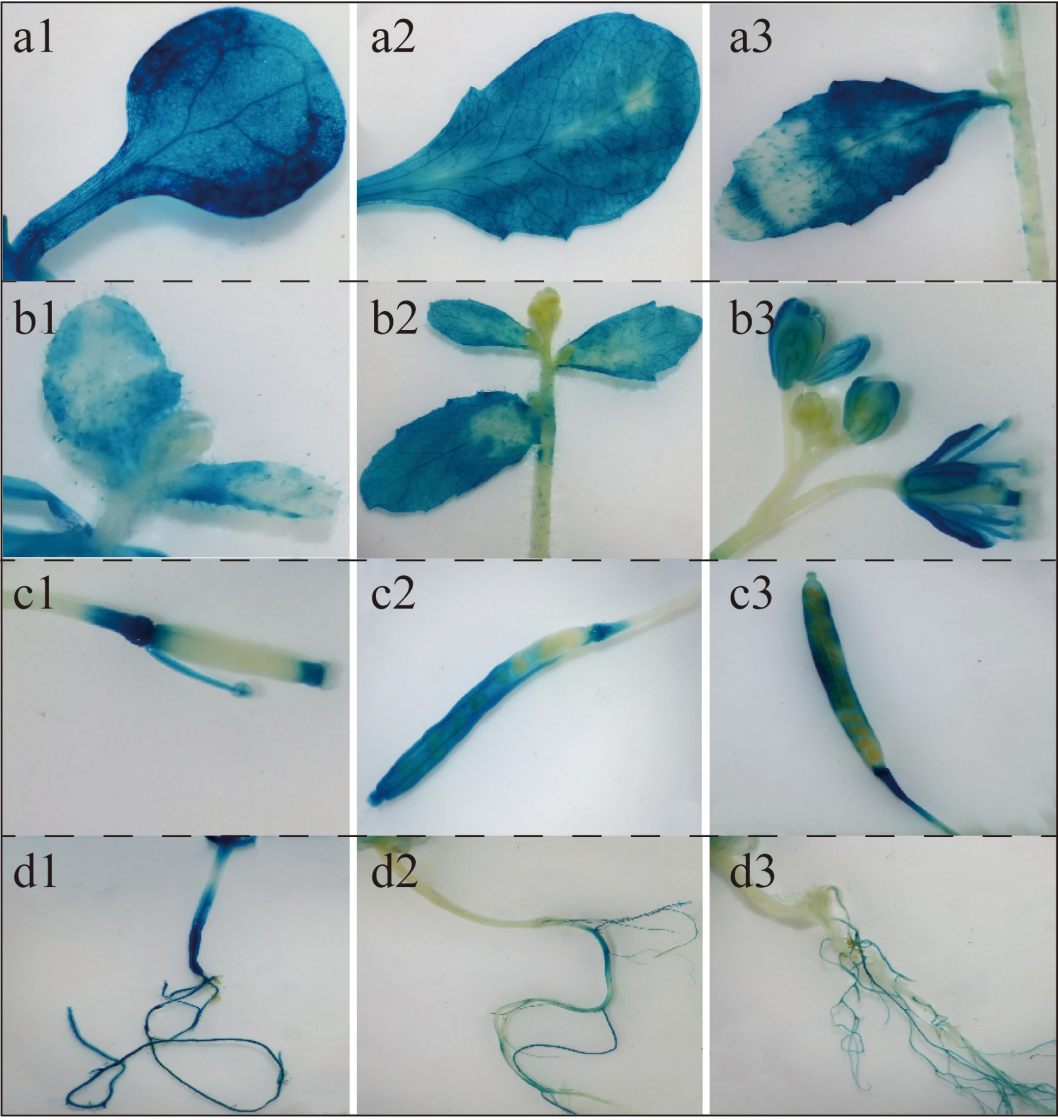
Localization of *BcGRP23* expression at different stages using pBcGRP23::GUS for β-glucuronidase (GUS) staining. **a1–a3**, cotyledons, rosette leaves, and cauline leaves; **b1–b3**, flowers, leaves, and stems in the bolting, bud, and flowering stages; **c1–c3**, pods in the pod setting, seed expansion, and seed maturity stages; **d1–d3**, roots in the bolting, flowering, and fruiting stages.

A chemical staining analysis was performed using *proBcGRP23-*GUS transformed *Arabidopsis* plants at various growth stages and organs. GUS staining was observed in the roots and leaves of the *proBcGRP23*::GUS transgenic lines throughout the growth period (**Figure 7, a1–a3, b1–b3, d1–d3**), with the darkest staining observed in the cotyledons (**Figure 7, a1**). During the bolting, bud, and flowering stages, a weak GUS stain was observed in the stems and growing points, whereas an intense GUS stain was observed in the petals and stamens of the 35S::GUS transgenic lines (**Figure 7, b1–b3**). The GUS gene was mainly expressed at the top of the silique during the podding stage, in the direct junction between the stalk and silique in the early stage, and then mainly in the pod (**Figure 7, c1**). The GUS stain was also observed in the stalk after the seeds were almost mature (**Figure 7, c2, c3**). An intense GUS stain was observed in the hypocotyl; however, the intensity became weaker with time. After anthesis, the GUS stain was undetectable (**Figure 7, d1–d3**).

### Effects of phytohormones on the expression of *GUS* driven by the *BcGRP23* promoter in transgenic *Arabidopsis*

**Figure 8:**
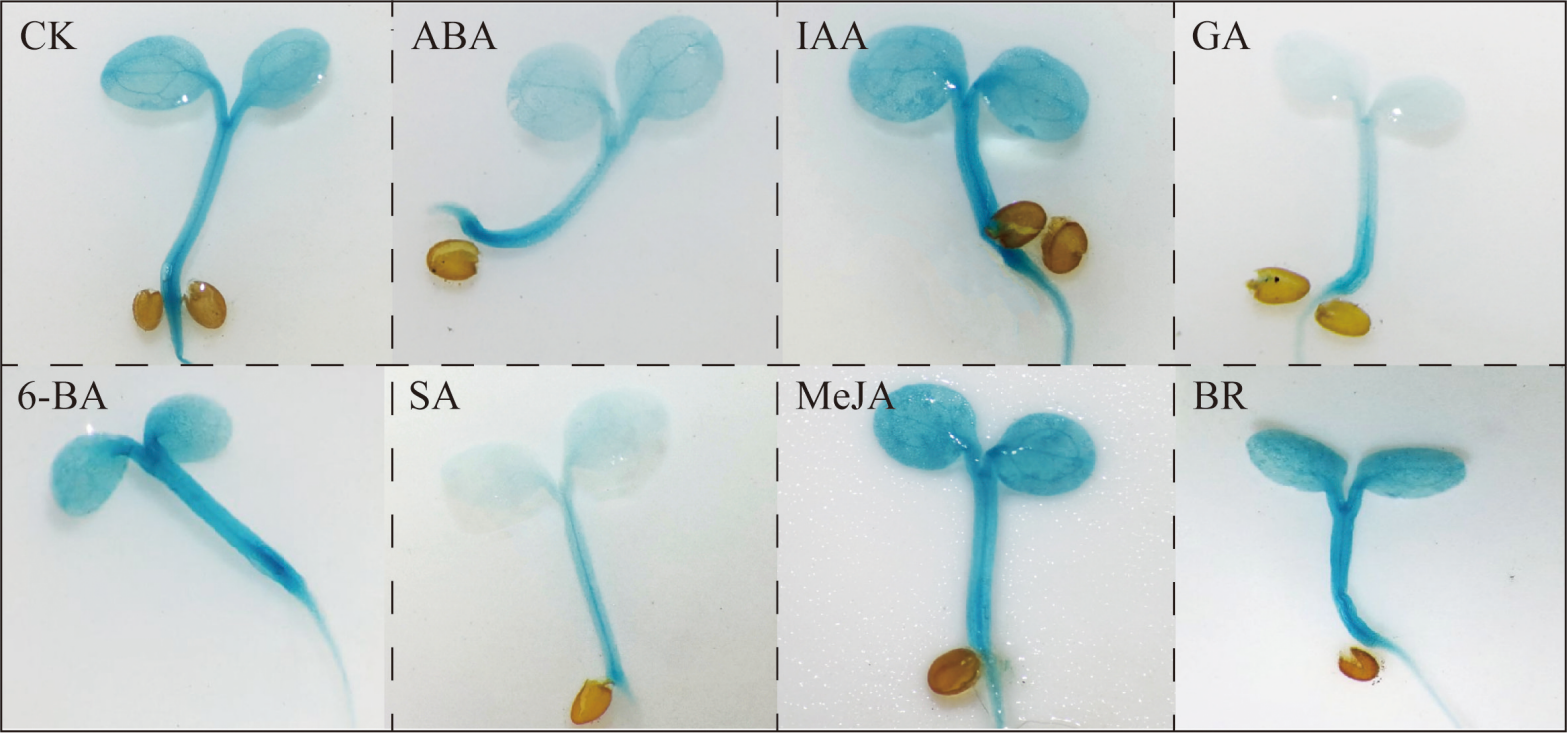
Patterns of β-glucuronidase (GUS) staining in *Arabidopsis* carrying p*BcProGRP23*::GUS and reporter constructs and treated with phytohormones.

To determine whether exogenous phytohormones regulate the transcription level of *BcGRP23*, histochemical analysis of GUS was performed on *ProBcGRP23*::GUS transgenic *Arabidopsis* seedlings in the cotyledon stage treated with 50 μM phytohormones (ABA, IAA, GA, 6-BA, SA, MeJA, and BR). Compared to the control plants (treated with water), the GUS stain became stronger in plants treated with IAA, 6-BA, MeJA, and BR, but it became lighter in plants treated with ABA, GA, and SA (**Figure 8**). There was no significant difference in the GUS staining intensity after 3 and 6 h of treatment. These results indicate that *BcGRP23* is involved in the regulation of various phytohormones.

### BES1 acts as a transcriptional activator of *BcGRP23*

**Figure 9:**
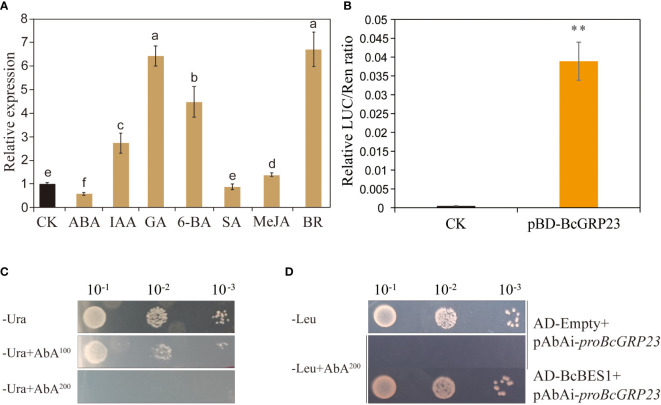
Analysis of the BES1 transcription factor that regulates *BcGRP23* promoter activity. **(A)** Effects of different hormones on the expression of *BcGRP23* in flowering Chinese cabbage. Different lowercase letters indicate significant differences at the p < 0.05 level. **(B)** Luciferase (LUC)/*Renilla* (REN) ratio indicating the relative luciferase activity in the dual-LUC assay. **Significant difference at the *p <* 0.01 level. **(C, D)** Yeast one-hybrid analysis. **(C)** Autoactivation of the *BcGRP23* promoters was tested on synthetically defined (SD) medium without Ura in the presence of aureobasidin A (AbA). *AbA^100^*, 100 ng/ml AbA; *AbA^200^*, 200 ng/ml AbA. **(D)** Interactions were determined on SD medium without Leu in the presence of 200 ng/ml AbA (−Leu+AbA^200^). (*a*) Negative control: AD-Empty+*BcGRP23* promoter.

To determine the effect of hormones on the transcription level of *BcGRP23* in flowering Chinese cabbage, the expression of *BcGRP23* was examined in 3-week-old seedlings treated with exogenous hormones. Compared with the control (treated with water), the transcription level of *BcGRP23* significantly increased after hormone (IAA, GA, 6-BA, MeJA, and BR) treatments in flowering Chinese cabbage, but significantly decreased after ABA treatment. Notably, the difference between the control and the SA groups was not significant, as shown in **Figure 9A**. The expression of *BcGRP23* has been reported to be maximal under BR treatment. Thus, the transcription level of *BcGRP23* is regulated by various hormones, particularly BR (**Figure 9A**). To assess the regulation of *BcGRP23* by the transcription factor BES1, using the *BcGRP23* promoter was verified in a yeast expression system. A 572-bp *BcGRP23* promoter containing three BES1-binding elements (CANNTG) was selected for the Y1H assay. The results showed that BES1 can bind to the *BcGRP23* promoter in a yeast expression system (**Figures 9C, D**). When the interaction of BcBES1 with the *BcGRP23* promoter was analyzed, the expression of the positive control (62-BES1-SK+pGreenII 0800-proBcGRP23-LUC), BES1, was found to result in a higher LUC/REN ratio than that of the negative control (**Figure 9B**). These results indicate that BES1 is a transcriptional activator of *BcGRP23* and enhances the activity of the *BcGRP23* promoter.

## Discussion

GRPs are part of a large and complex family sharing the common feature of a (Gly)nX repeat-rich glycine domain that may be involved in protein–protein interactions, RNA binding, and nuclear targeting (Giarola et al., 2016). GRPs are involved in multiple independent physiological processes, such as cell wall building, growth and development, and stress resistance in plants (Czolpinska and Rurek, 2018). In this study, we investigated the role of *BcGRP23* in the flowering Chinese cabbage. Previously, transient expression in the onion epidermis verified the localization of *BcGRP23* in the cell wall; it contained (GGX)*_n_* repeats that are specific to class I GRPs. These results are consistent with a previous study showing thDat *AtGRP23* was located in the cell wall and contained more than 60% of the total amino acids with glycine (Park et al., 2008). *BcGRP23* was preliminarily determined to be a class I GRP.

According to many studies, GRPs have tissue and spatiotemporal specificity in plants; however, their expression is strongly suppressed in matured tissues (Lei and Wu, 1991). *OsGRP-2* was found to be barely expressed at the seedling stage in rice, but was significantly expressed after the seedling stage. Thereafter, its expression was found to gradually increase, reaching the highest level at the jointing stage and then gradually decreasing at the booting stage (Liu et al., 2003). In this study, the expression of *BcGRP23* was found to significantly vary in the different stages and tissues of flowering Chinese cabbage, as shown in **Figure 3**. The expression level of *BcGRP23* was the highest in the leaves in the cotyledon stage, the flowers in the cotyledon stage, and the flowers and seeds in the flowering stage. Furthermore, its expression was slightly lower in the roots throughout the growth cycle and in the stems and leaves in the flowering stage (**Figure 3**). These findings are consistent with the predicted results of the *BcGRP23* promoter sequence. Numerous tissue-specific *cis*-elements, such as leaves, flowers, and seeds, were found in the *BcGRP23* promoter (**Supplementary Table S1**). Moreover, *BcGRP23* was recognized to have spatiotemporal specificity in the aboveground parts of flowering Chinese cabbage.

The whitening phenomenon in plant tissues is mainly caused by a significant reduction in chlorophyll content. However, this phenomenon may have occurred due to inhibition of the biosynthesis of chlorophyll, or acceleration of metabolism, and the abnormal development of the tissue structure of chloroplast (Thomas et al., 2001). Based on previous studies, SGR1 is a positive regulator of chlorophyll degradation during *Arabidopsis* senescence (Gao et al., 2016). Under natural senescence and dark-induced conditions, the *sgrl-1* mutant *Arabidopsis* displayed a stay-green phenotype, whereas *SGR1*-overexpressing *Arabidopsis* leaves exhibited an early yellowing trait (Sakuraba et al., 2013; Li et al., 2017). In the present study, *BcGRP23*-silenced plants displayed whitening of the stems and petioles as the levels of Chl *a* and Chl *b* decreased (**Figure 4**). These findings are consistent with those of a previous study that revealed that SGRl controls the leaf color in *Arabidopsis*. Accordingly, we speculate that *BcGRP23* is a previously unknown regulator of chlorophyll degradation and biosynthesis (Li et al., 2017).

*PAO* is involved in chlorosis and can catalyze the opening of the chlorophyll porphyrin ring to form a quaternary linear pyrrole derivative; the absence of *PAO* leads to the stay-green trait in plants (Hörtensteiner, 2009). During ripening, *PAO* interacts with the chlorotic gene *SGR*, and its activity on demagnesium chelatase gradually increases, with the highest level achieved at the ripening stage, causing rapid degradation of chlorophyll and chlorosis in plants (Costa et al., 2002; Hörtensteiner, 2013). The proteins translated by *NYCI* and *NOL* colocalize on the thylakoid membrane of the chloroplast to form a Chl *b* reductase complex, which catalyzes the degradation of Chl *b* during the mature stage, resulting in rapid chlorophyll degradation and chlorosis in plants (Sato et al., 2009).

The expression levels of the genes related to the chlorophyll biosynthesis and degradation pathways in *BcGRP23*-silenced plants were significantly different from those in WT plants. In fact, the expression levels of five chlorophyll degradation genes, namely, *BcPAO*, *BcPPH*, *BcNYC1*, *BcRCCR*, and *BcSGR1*, were significantly increased. The expression levels of five chlorophyll biosynthesis-related genes—*BcPORA*, *BcPORB*, *BcPORC*, *BcCAO*, and *BcCHLD*—were also upregulated, but to a lesser extent than those of the chlorophyll degradation genes (**Figure 5**). Notably, changes in these genes led to a reduction in chlorophyll content in the petioles of flowering Chinese cabbage, which may be responsible for the whitening of the petioles and stems. The expression levels of the chlorophyll degradation genes were significantly reduced in plants overexpressing *BcGRP23*; however, the expression levels of some of the chlorophyll biosynthesis-related genes were not found to differ significantly (**Figure 6**). Overall, the increase in *BcGRP23* had a greater effect on the chlorophyll biosynthesis genes than on the chlorophyll degradation genes, which might be the reason for the increased chlorophyll content in overexpressed plants.

A total of 15 core *cis*-elements (TATABOX) responsible for initiating transcription were identified, including 13 elements (CAATBOX) that regulate the transcription initiation frequency and two initiation factors (INRNTPSADB) in the *BcGRP23* promoter region. These elements play a role in the promotion of transcription, which suggests that the *BcGRP23* promoter has strong activity (Butler and Kadonaga, 2001). Notably, the *BcGRP23* promoter sequence also contained hormone response elements (ethylene, cytokinin, abscisic acid, brassinolide, GA, and MeJA), indicating that *BcGRP23* is associated with the hormone signaling pathways and might interact with these hormones during plant growth and development.

In *Arabidopsis*, drought and salt stress were found to upregulate the transcription level of *AtGRP1*, but to downregulate the transcription levels of *AtGRP4* and *AtGR7*. *AtGRP7* was also found to be inhibited by endogenous ABA and osmotic stress (Cao et al., 2006). The expression of *NtGRP1* and *NtGRP3* in tobacco can be disrupted by high salinity, low temperature, drought, high temperature, and mechanical damage; however, their expression levels are low (Lee et al., 2009; Xuan et al., 2010). *HvGRP2* and *HvGRP3* in barley are induced by fungal pathogens and are regulated by MeJA and ethylene (Molina et al., 1997). Here, several ionic stressors (copper, phosphorus, and sulfur), drought, salicylic acid, injury-specific stress, salt stress, low-temperature stress, and other stress-related regulatory elements were found, indicating that *BcGRP23* may be involved in the regulation of various abiotic stresses. A total of 64 light regulatory elements, including I-BOX, REALPHALGLHCB21, GT1 motif, -10PEHVPSBD, and chlorophyll gene-related regulatory elements (3 CPBCSPOR and 26 GATABOX), were also found in the *BcGRP23* promoter region, which suggests that the expression of *BcGRP23* was affected by light and is related to the chlorophyll cycle (**Supplementary Figure S1**). The *BcGRP23* promoter was transformed into *Arabidop1sis*, and GUS histochemical staining was carried out using transgenic *Arabidopsis* at different time points in different plant parts. GUS staining was observed in the roots and leaves throughout the growth period. Notably, only a small amount of GUS stain was observed at the growth points and stems in the bolting, bud, and flowering stages (**Figure 7**). The GUS activity of *BcGRP23* was inconsistent between the tissue expression in flowering Chinese cabbage and the heterologous expression of the *BcGRP23* promoter in *Arabidopsis*; species differences may have influenced the gene expression.

Plant hormones play an important role as signaling molecules in plant development and in response to stressful environments (Ton et al., 2009). During response to biotic stress, the SA and JA hormone signaling pathways play a vital role in regulating the expression of disease resistance genes (He et al., 2016). IAA and ABA are vital hormones involved in plant growth and development (Emenecker and Strader, 2020), while BR and ABA act as stress hormones and play key roles in plant stress resistance (Bari and Jones, 2009). In the present study, the GUS stain became more intense after treatments with IAA, 6-BA, MeJA, and BR, but became lighter after treatments with ABA, GA, and SA. Analogously, normal-growing flowering Chinese cabbage was treated with the hormones IAA, GA, 6-BA, MeJA, and BR. Compared to the control, the transcription level of *BcGRP23* increased significantly after 6 h, with the most significant increase observed with BR treatment, and decreased following treatment with ABA. In conclusion, *BcGRP23* is regulated by various hormones, especially BR (**Figure 8**). Thus, *BcGRP23* is strongly correlated with plant hormones, resulting in a significant increase in its transcript levels (**Figure 9A**).

It is known that the application of BR regulates plant growth, the net photosynthetic rate, and the antioxidant system capacity. Epi-brassinolide (EBL) treatment significantly increased the Chl *a* and Chl *b* contents, photosynthetic rate (*P*_n_), and water use efficiency in tomato under Cd stress, thereby alleviating the inhibitory effects of Cd (Guo et al., 2018). BR-insensitive mutants of *Arabidopsis* and *Brassica* exhibited dwarfing, dark green leaves, late flowering, and male sterility (Guo et al., 2018). Brassinazole-resistant 1 (BZR1) and BES1 are considered important transcription factors in the BR signal transduction pathway that can bind to BR-responsive genes and play a regulatory role (Lee et al., 2015). The BR-responsive transcriptome data revealed that the promoter regions of the BR-inducible genes are rich in E-box (CANNTG) binding sites, while those of the BR-repressor genes are rich in BRRE *cis*-acting elements (Guo et al., 2018). In this study, eight E-boxes were found in the *BcGRP23* promoter. In addition, the BR transcription factor, BES1, could bind to the *BcGRP23* promoter in the yeast expression system (**Figures 9C, D**). The dual-luciferase assay revealed positive regulation of the *BcGRP23* promoter by the BR transcription factor, BES1 (**Figure 9B**). Overall, our findings indicate that BR may respond to various stresses by regulating the transcription of *BcGRP23* and activating different physiological and molecular mechanisms to promote plant growth and development and improve plant stress tolerance. The response of GRPs to environmental stress in plants has been widely explored over the past few decades. The findings of this study provide new insights into the involvement of GRPs in the response to environmental stress, growth, and the developmental mechanisms of flowering Chinese cabbage.

## Conclusions

In this study, we preliminarily determined the biological functions and potential regulatory pathways of *BcGRP23* during development, interaction with hormones, and adverse stress in flowering Chinese cabbage and *Arabidopsis* mutants. *BcGRP23* localized on the cell wall was found to regulate the expression of chlorophyll degradation-related genes, thus affecting chlorophyll content. *BcGRP23* was highly expressed in the leaves, flowers, and seeds at the cotyledon stage; however, its expression levels gradually decreased in the leaves and stems with the growth of flowering Chinese cabbage. *BcGRP23* exhibited a significant response to the exogenous hormones IAA, GA, 6-BA, MeJA, and BR and showed enhanced transcript levels. The Y1H assay and relative luciferase activity revealed a potential interaction between *BcGRP23* and BES1. Collectively, our findings provide new insights into the role of GRP in the hormonal regulatory network and stress response in flowering Chinese cabbage. Such findings provide a fundamental basis for further studies on the *GRP* gene family and will be helpful for crop genetic modification.

## Data availability statement

The original contributions presented in the study are included in the article/**Supplementary Material**. Further inquiries can be directed to the corresponding authors.

## Author contributions

SZ, KC, and WS conceived and designed the experiments. SZ, KC, and SY conducted the experiments and analyzed the data. SZ and KC prepared the manuscript. AA and YW participated in the experiments and revised the manuscript. SS, WS, and RC offered experimental guidance. All authors contributed to the article and approved the submitted version.

## Funding

This study was supported by the National Natural Science Foundation of China (32072656); the Key-Area Research and Development Program of Guangdong Province, China (2020B0202010006); the Guangdong Provincial Special Fund for Modern Agriculture Industry Technology Innovation Teams (2022KJ131); and the China Agriculture Research System of MOF and MARA.

## Conflict of interest

The authors declare that the research was conducted in the absence of any commercial or financial relationships that could be construed as a potential conflict of interest.

## Publisher’s note

All claims expressed in this article are solely those of the authors and do not necessarily represent those of their affiliated organizations, or those of the publisher, the editors and the reviewers. Any product that may be evaluated in this article, or claim that may be made by its manufacturer, is not guaranteed or endorsed by the publisher.
