# Supplementary material for: BcGRP23: A novel gene involved in the chlorophyll metabolic pathway that is activated by BES1 in flowering Chinese cabbage
Source: Front Plant Sci. 2022 Oct 19;13:1010470. doi: 10.3389/fpls.2022.1010470 (PMC9639331; doi:10.3389/fpls.2022.1010470)
Supplement: Supplementary file 1 [file DataSheet_1.pdf]

Supplementary Material

A

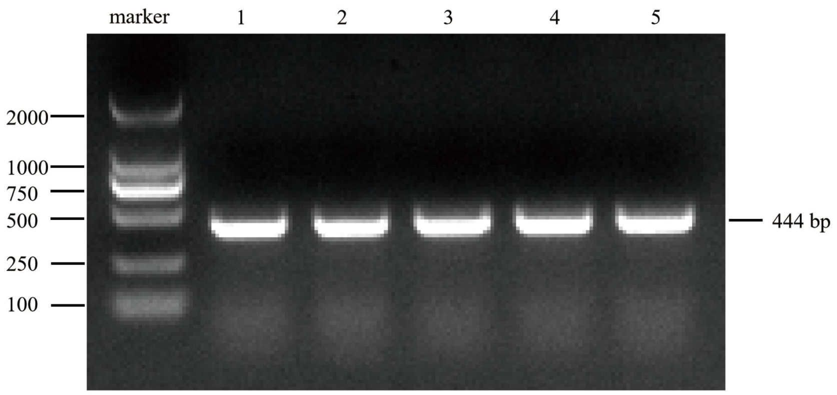

B

ATGGCAATAAGGTGTTTAGCCGTGTTCCTCGTTGCTCTCACTGTGGCTCAGTCCGTCAGTGCCACAAGACCGGCCCCGGCCAAAAATGTCGGA
M A I R C L A V F L V A L T V A Q S V S A T R P A P A K N V G
GCTGGCCTTGATGACCAAAAGAACTTTGTTGCGTTTGCTGGGATCGGTGGAGCTGCTGGAGTTGGTGGTGTTGGTGGTGTCGGAACAGGTCTT
A G L D D Q K N F V A F A G I G G A A G V G G V G G V G T G L
GGTGGTGTAGCCGGTGGAGTCGGTGGTGTCGCCGGAGTTTTGCCTGTAGGCGGAGTAGGAGGAGGAATTGGTGGTTTAGGAGGTGTAGGTGGT
G G V A G G V G G V A G V L P V G G V G G G I G G L G G V G G
CTCGGTGGTGGTTCAGGTCTCGGTGGTGGAATCGGTGGAATTGGTGGTGGTTCAGGTATTGGTGGTGGTGTCGGTGGACTCGGAGGTGTAGGC
L G G G S G L G G G I G G I G G G S G I G G G V G G L G G V G
GGTCTAGGCGGAGTTGGTGGCTTGGGAGGAATTGGCGGTGGAAGCGATTGCGGTGGTCTCATTCACCCTTGA
G L G G V G G L G G I G G G S D C G G L I H P *

Supplementary Figure 1. Cloning of the *BcGRP23* gene. **(A)**, PCR amplification electropherogram. DNA marker: DL2000; 1-5 are the largest ORF sequence fragments of BcGRP23. **(B)**, BcGRP23 amino acid sequence.

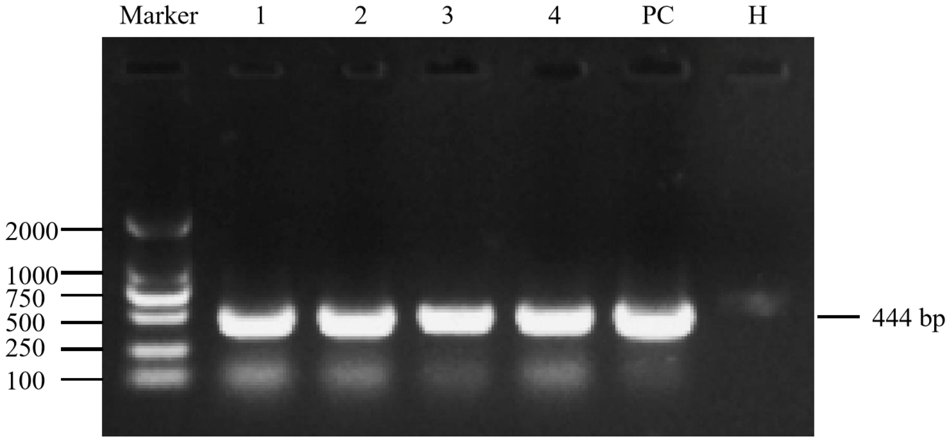

Supplementary Figure 2. Bacterial liquid PCR detection of pBI121-BcGRP23-GFP.

Note: Maeker: DL2000 ; 1~4: pBI121-BcGRP23-GFP; PC: positive control; H: negative control. The gel size is 444bp.

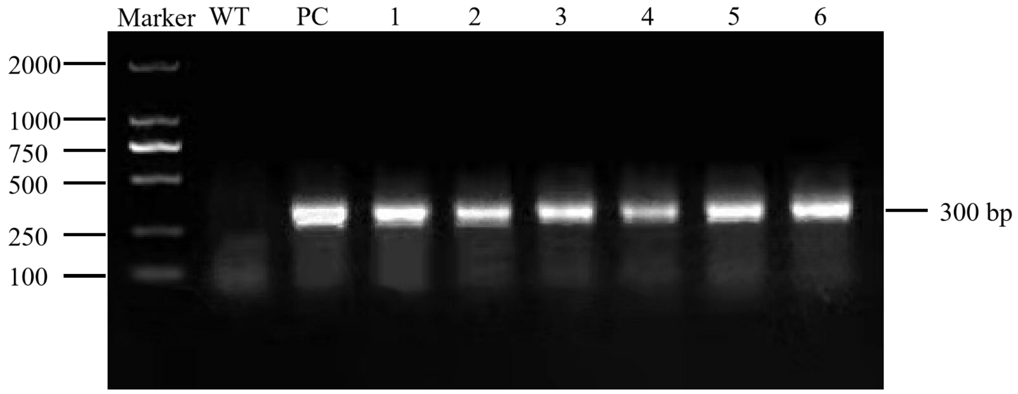

Supplementary Figure 3. Bacterial liquid PCR detection of pTRV2-BcGRP23.

Note: Maeker: DL2000; 1~6: pTRV2-BcGRP23; PC: positive control; WT: negative control; The gel band size is 300 bp.

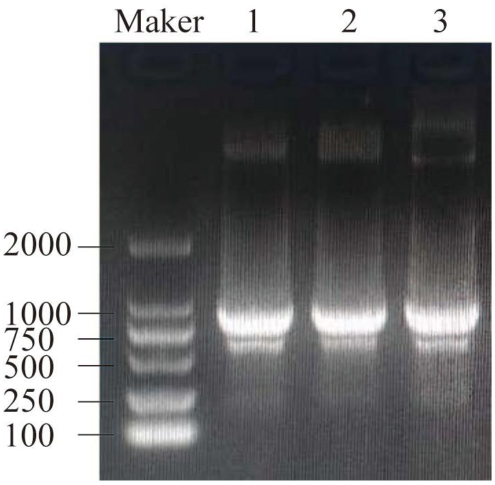

Supplementary Figure 4. Bacterial liquid PCR detection of CAMBIA1391-ProBcGRP23

Note: Maeker: DL2000; 1~3: pCAMBIA1391-ProBcGRP23.

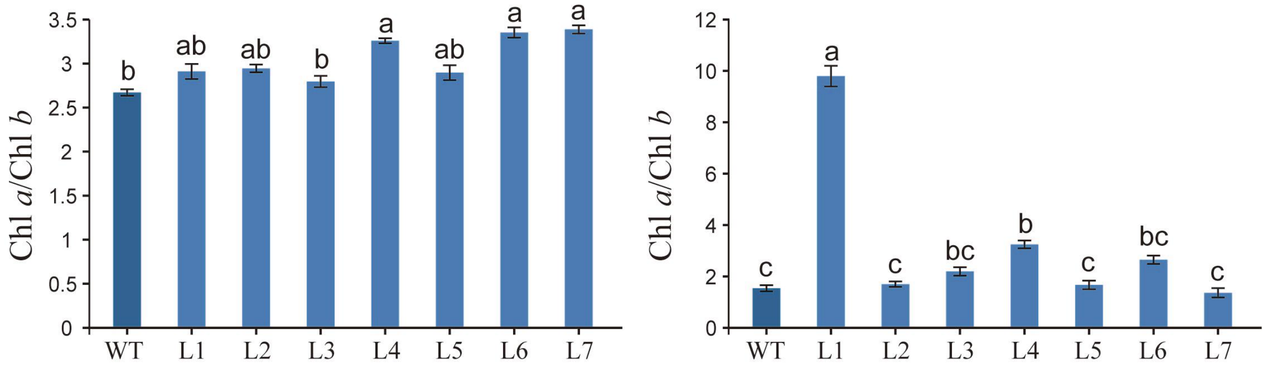

Supplementary Figure 5. The ratio of Chl*a* and Chl*b* (Chl*a*/*b*) in leaves (A) and petioles (B) of BcGRP23 knockdown lines. L1-L7 represent different BcGRP23 knockdown lines. Error bars represent standard errors. Different lowercase letters indicate significant differences at the $P < 0.05$ level.

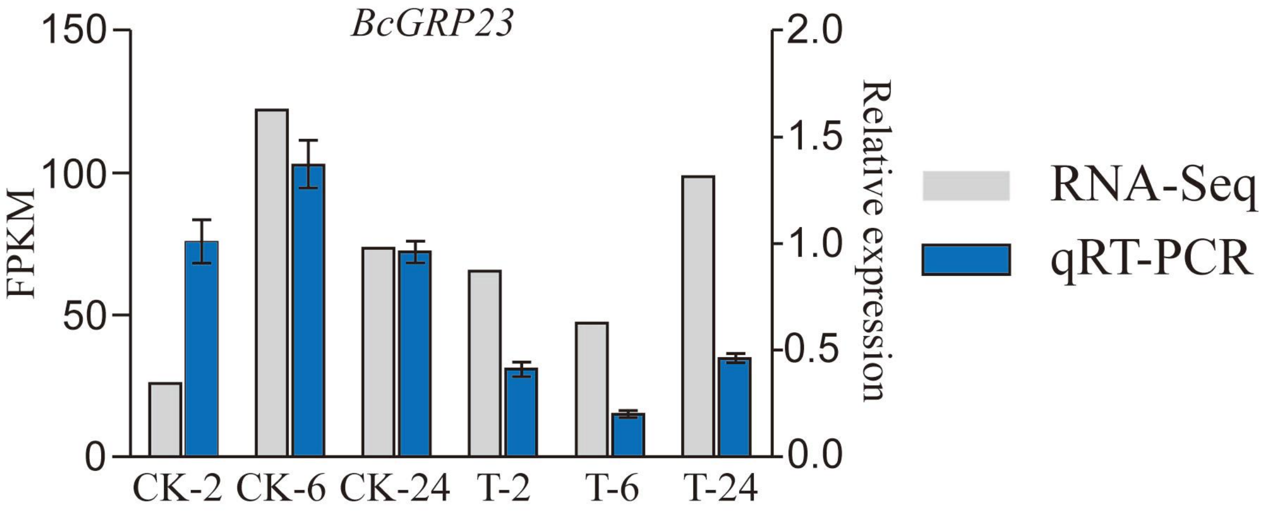

Supplementary Figure 6. The expression level of *BCGRP23* gene after PCZ (Propiconazole) treatment (83.3 mg/L). The left Y axis represents RNA-Seq results and the right Y axis represents the results of qRT-PCR. T-2: after 2 hours of treatment; T-6: after 6 hours of treatment; T-24: after 24 hours of treatment.

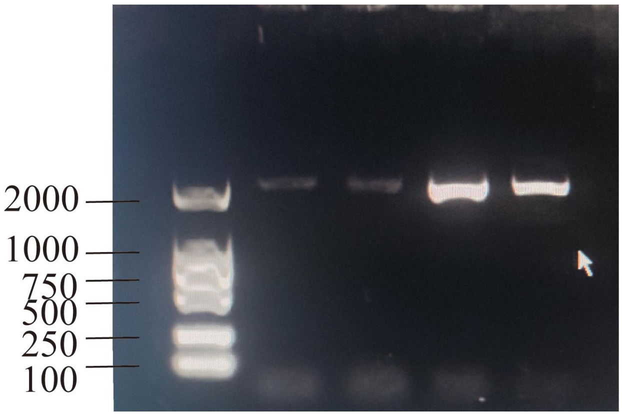

Supplementary Figure 7. Bacterial liquid PCR detection of pABAI-*ProBcGRP23*. The correct band size is: 1350 bp + insert size.

Supplementary Table 1. Analysis of cis-acting elements in the *BcGRP23* promoter

Name	Quantity	Sequence	Function
-10PEHVPSBD	3	TATTCT	light-responsive element
ABRELATERD1	1	ACGTG	ABA-responsive elements
ABRERATCAL	1	MACGYGB	abscisic acid-responsive element
ACGTATERD1	6	ACGT	drought-responsive element
ARR1AT	13	NGATT	cytokinin-responsive element
ASF1MOTIFCAMV	1	TGACG	salicylic acid-, auxin-responsive element
BIHD1OS	4	TGTCA	disease resistance-related response element
CAATBOX1	13	CAAT	elements that regulate transcription initiation frequency
CACTFTPPCA1	32	YACT	mesophyll-specific regulatory elements
CARGCW8GAT	11	CWWWWWWWWG	self feedback element
CATATGGMSAUR	4	CATATG	auxin-responsive element
CCAATBOX1	8	CCAAT	transcription factor associated with flowering gene transcription
CPBCSPOR	3	TATTAG	chlorophyll gene-related response elements
CURECORECR	12	GTAC	copper-inducible expression element
DOFCOREZM	15	AAAG	DOF gene binding site
DPBFCOREDCDC3	1	ACACNNG	abscisic acid-responsive element
EBOXBNNAPA	8	CANNTG	brassinolide, a seed development regulatory element
EECCRCAH1	2	GANTTNC	drought and osmotic stress response elements
ELRECOREPCRP1	1	TTGACC	damage specific responsive element
ERELEE4	2	AWTTCAAA	ethylene-responsive element
GATABOX	26	GATA	light regulation, chlorophyll a/b binding element

GT1CONSENSUS	22	GRWAAW	light-regulated GT-1 factor conserved binding site
GT1CORE	2	GGTTAA	light-responsive element
GT1GMSCAM4	2	GAAAAA	salt regulatory element
GTGANTG10	8	GTGA	pollen late gene promoter element
IBOX	2	GATAAG	light-responsive element
IBOXCORE	15	GATAA	light-responsive element
INRNTPSADB	2	YTCANTYY	initiation factor Inr element
LTRE1HVBLT49	2	CCGAAA	low temperature response element
MYB1AT	12	WAACCA	ABA, Dehydration response element
MYB2CONSENSUSAT	2	YAACKG	drought response element
MYBCORE	4	CNGTTR	dehydration, Light regulation related element
MYBCOREATCYCB1	4	AACGG	light regulation related element
MYBPLANT	2	MACCWAMC	ethylene,flower organ tissue specific element
MYCCONSENSUSAT	8	CANNTG	cold stress specific element
NODCON2GM	3	CTCTT	root tissue specific expression element
NTBBF1ARROLB	2	ACTTTA	auxin induction related element
OSE2ROOTNODULE	3	CTCTT	junction-specific element
P1BS	4	GNATATNC	phosphorus deficiency response element
POLASIG1	6	AATAAA	polyadenylation site
POLLEN1LELAT52	8	AGAAA	floral organ-specific element
PYRIMIDINEBOXOSRAMY1A	1	CCTTTT	gibberellin response element
RAV1AAT	6	CAACA	SA signaling pathway-related cis-element

REALPHALGLHCB21	7	AACCAA	light regulatory element
REBETALGLHCB21	3	CGGATA	light regulatory element
RHERPATEXPA7	2	KCACGW	root hair specific element
ROOTMOTIFTAPOX1	30	ATATT	root specific regulatory element
SEF1MOTIF	2	ATATTTAWW	enhancer promoter
SEF3MOTIFGM	1	AACCCA	binding site for embryo-specific protein SEF3
SEF4MOTIFGM7S	3	RTTTTTR	binding site for embryo-specific protein SEF4
SREATMSD	2	TTATCC	sugar repressive element
SURECOREATSULTR11	2	GAGAC	sulfur deficiency response element
TAAAGSTKST1	4	TAAAG	guard cell specific gene expression element
TATABOX2	4	TATAAAT	promoter core element
TATABOX3	2	TATTAAT	promoter core element
TATABOX4	3	TATATAA	promoter core element
TATABOX5	6	TTATTT	promoter core element
TBOXATGAPB	2	ACTTTG	light regulatory element
WBBOXPCWRKY1	2	TTTGACY	defense response gene expression regulatory element
T/GBOXATPIN2	1	AACGTG	methyl jasmonate inducible element element.
WBOXATNPR1	5	TTGAC	salicylic acid inducible element
WBOXHVISO1	2	TGACT	sugar response element
WBOXNTERF3	4	TGACY	injury-related element
WRKY71OS	9	TGAC	gibberellin-related element

Supplementary Table 2. List of primers used in this study

Primer name.	Primer sequence (5'-3').	
GAPDH	F:CAGGTTTGGAATTGTCGAGG<br>R:GAGCTGTGGAAGCACCTTTC	Internal reference gene
BcGRP23-DL	F:GCTGGCCTTGATGACCAAAA<br>R:CTACTCCGCCTACAGGCAAAA	*BcGRP23* for qRT-PCR
BcGRP23-CDS	F:ATGGCAATAAGGTGTTTAGCC<br>R:TCAAGGGTGAATGAGACCAC	Full-length amplification of *BcGRP23* CDS.
pBI121-BcGRP23	F:CACGGGGGACTCTAGAATGGCAATAAGGTGTTTAGCC<br>R:TCCTTTACCCATCCCGGGAGGGTGAATGAGACCAC	*Xba* I and *Sma* I
pCAMBIA1391-ProBcGRP23	F:CCGGCGCGCCAAGCTTTGATATTTACAGTTTAGCAA<br>R:GGGGATCCGTCGACCTGCAGCATTGGGAGGTTTATATAGGA	*Hin*d III and *Pst* I
pTRV2-BcGRP23	F:CACGGGGGACTCTAGACCAATACCTGAACCACCACC<br>R:TCCTTTACCCATCCCGGGTGTGGCTCAGTCCGTCAGTG	*Xba* I and *Sma* I
GUS	F:TAATGTTCTGCGACGCTCACA<br>R:ATACCTGTTCACCGACGACG	*GUS* gene for qRT-PCR
pGreen Ⅱ -62-SK-BES1	F:GGCCGCTCTAGAACTAGTGGATCCATGACAGCATCAGGAG<br>R:ATCGATAAGCTTGATATCGAATTCTTAGCCGCGTCCTTTAGC	*BamH* I and *EcoR* I
pGreen Ⅱ -0800-LUC-ProGRP23	F:CACTATAGGGCGAATTGGGTACCTGATATTTACAGTTTAGCAA<br>R:TTTATGTTTTTGGCGTCTTCCATC	*Kpn* I and *Nco* I

	ATTGGGAGGTTTATATAGGA	
pABAi-ProGRP23	F:CTTGAATTCGAGCTCGGTACCTA AGGTAACATGAGATACATATC R:AGCACATGCCTCGAGGTCGACG CATAAGTTACGCAAAAGGC	*Knp* I and *Sal* I
pGADT7-BcBES1	F:GGAGGCCAGTGAATTCGAGAAT GACAGCATCAGGAGG R:CGAGCTCGATGGATCCCCTCTTA GCCGCGTCCTTTAG	*EcoR* I and *BamH* I

Supplementary Table 3. List of primers for genes related to chlorophyll synthesis and degradation in flowering Chinese cabbage

Gene name	Primer sequence (5'-3').
BcPORA	F:CGGCGATGATAGATGGAGG
	R:AAGAGTGAACGGAAGAGAGGAATA
BcPORB	F:CGATGATTGATGGAGGAGATT
	R:GCAACCAGGGTAAAGCGAAG
BcPORC	F:AAATCTGACTATCCATCAAAACG
	R:GCTCCATCATACTCTCCTCCA
BcCHLD	F:GCACACCAGCAAACTCTCTCA
	R:TCTTCGTCCTTATCACCTCCG
BcCAO	F:TGCGACAGAATCAGACAGACC
	R:AAGCAACGGGATACCAAAAGT
BcPAO	F:CAGCTTCAGCGACACTCACC
	R:TCGCCGTGCTCTTCTTCGAT
BcPPH	F:TATCTGATGCGCGGGTGGAT
	R:TTCCCGACCAATGCTGGACT
BcSGR1	F:GTTGGGGTCCGCTTTGGGAA
	R:AATCGAGCTAACCTGCGGGA
BcNYC1	F:TGCTTCTCCGGGCATGGTTC
	R:CTGTCCGCTTCCGCTGCATA
BcRCCR	F:CTCGACCTCCCTCATCGCAA
	R:CGGACAAAGAGAGACGGCGA

frontiers
